# Self-Diffusion of Ge in Amorphous Ge_*x*_Si_1–*x*_ Films Studied In Situ by Neutron Reflectometry

**DOI:** 10.1021/acsmaterialsau.4c00046

**Published:** 2024-07-23

**Authors:** Erwin Hüger, Jochen Stahn, Harald Schmidt

**Affiliations:** † Institute of Metallurgy, Solid State Kinetics Group, Clausthal University of Technology, Clausthal-Zellerfeld 38678, Germany; ‡ Clausthal Center for Materials Technology, Clausthal-Zellerfeld 38678, Germany; § Center for Neutron and Muon Sciences, Paul Scherrer Institute, Villigen PSI 5232, Switzerland

**Keywords:** neutron reflectometry, amorphous films, germanium silicide, self-diffusion, activation energy, in situ measurement

## Abstract

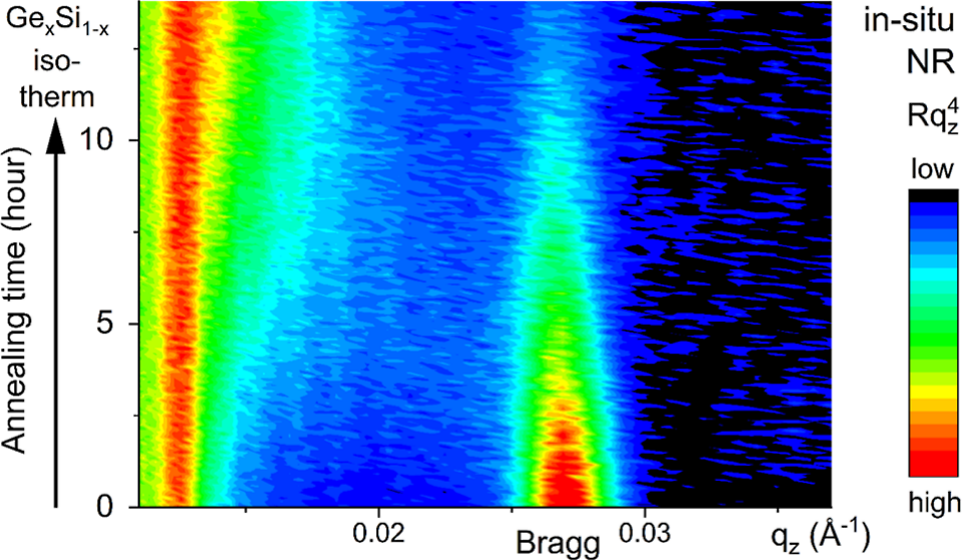

Ge_*x*_Si_1–*x*_ alloys are gaining renewed interest for many applications in electronics and optics, especially for miniaturized devices showing quantum size effects. Point defects and atomic diffusion play a crucial role in miniaturized and metastable systems. In the present work, Ge self-diffusion in sputter deposited amorphous Ge_*x*_Si_1–*x*_ alloys is studied in situ as a function of Ge content *x* = 0.13, 0.43, 0.8, and 1.0 by neutron reflectometry. The determined Ge self-diffusivities obey the Arrhenius law in the investigated temperature ranges. The higher the Ge content *x*, the higher the Ge self-diffusivity at the same temperature. The activation enthalpy decreases with *x* from 4.4 eV for self-diffusion in pure silicon films to about 2 eV self-diffusion in Ge_0.8_Si_0.2_ and Ge. The decrease of the activation enthalpy for amorphous Ge_*x*_Si_1–*x*_ is similar to the case of crystalline Ge_*x*_Si_1–*x*_. Possible explanations are discussed.

## 1 Introduction

Amorphous germanium–silicon (Ge–Si) solid solutions are an interesting class of materials from both a fundamental and technological point of view. Although Ge–Si is a long-known alloy, it is classified as an emerging material due to the renewed interest in a variety of applications, including solar cells, thermoelectrics, photonics, spintronics, quantum computing,^1−5^ and as an active material^6,7^ in negative electrodes of lithium-ion batteries (LIBs).^6−14^ For the last case, silicon, germanium, and, hence Ge–Si solid solutions react with lithium via an alloying/dealloying process that stores and delivers on average ten times more of Li^+^ charge per atom than graphite, the anode material in commercial LIBs.^6−12^

The interest in Ge–Si alloys can be explained by the fact that although germanium and silicon are similar semiconductors due to their valence isoelectronicity, they also have some differences that become important. Germanium has a slightly larger lattice constant and a more metallic bonding character than its counterpart silicon, which can induce strain in Ge–Si heterostructures and a higher atomic, electron and hole mobility. The higher electronic and ionic conductivity (Li diffusivity) together with the more metallic bonding character, is thought to be beneficial for fast and long-cycle stability during LIB operation.^8−13^

Planar Ge, Si, Ge/GeSi and Si/GeSi heterostructures with layer thicknesses between 15 and 500 nm are systems of choice not only for the fabrication and study of solar cells, but also for the study of carrier mobility in quantum technologies.^2−5^ For the latter case confinement and quantum size effects (QSE) play a decisive role.^15−20^

Beneath the crystalline modifications, the materials are also stable in the amorphous state. Interestingly, the amorphous structure can improve device functionality compared to its crystalline counterpart. The amorphous network is seen as a solution to the disadvantages of crystalline Ge–Si in LIB operation.^7^ It is also relevant for low-cost fabrication of solar cells because the optical bandgaps match the full solar radiation spectrum.^21−23^ Ultrathin amorphous semiconductor heterostructures can improve the performance of solar cells by broadening the effective band gap, increasing the effective mass separation and the open circuit voltage.^17−20^

For all of these applications, atomic diffusion is important for long-term stability, especially for short length scales in confined regions (e.g., ultrathin films and quantum dots) required to achieve the desired QSE.^15^ In order to improve the performance of micro- and nanostructures, it is important to understand mass transport. In general, point defects in semiconductors and their manipulation by doping are the fundamental reason for the usefulness of this class of materials.^24,25^ Direct experimental characterization of defects like vacancies is experimentally challenging.^24^ Since self-diffusion takes place via point defects, the study of this phenomenon gives information on the nature and properties of point defects in semiconductors. This work contributes to the knowledge increase of Ge–Si nanostructures by measuring self-diffusion in amorphous Ge–Si thin films.

Atomic diffusion has been routinely investigated in the past by radiotracer diffusion studies^26−28^ with the disadvantage of using radioactive material and performing depth profiling with mechanical sectioning and limited depth resolution.^28^ A more recent development uses ion-beam sputtering for sectioning.^29^ Another disadvantage of the radiotracer method is the inability to measure diffusion in situ, i.e. during heat treatment without cooling the sample. This also applies to the use of stable tracer isotopes and depth profiling with secondary ion mass spectrometry.^30−43^ The in situ measurement of diffusivities has the advantage of a significant reduction in experimental time and error limits due to the omission of heating/cooling steps, and the identification of time-dependent processes.^44^ Nondestructive methods are required to perform such measurements.

Neutron reflectometry (NR)^41,44−59^ is such a nondestructive method. During the last years our group used and developed the method of NR in order to monitor atomic diffusion in situ or in-operando and in real time.^44,53−57,57−59^

With respect to self-diffusion, studies on amorphous semiconductors are rare. This is mainly due to the metastable^60^ or even unstable^61^ state of the amorphous structure. The diffusivity has to be determined on short lengths scales to avoid averaging over different transient metastable states and to avoid unwanted crystallization. NR experiments are capable of measuring small diffusion lengths down to 1 nm^44,45,48,49^ and thus in transient states.^48^ NR experiments to measure self-diffusion in amorphous germanium and silicon were successfully carried out by our group.^44,50,51,57^ The present study reports on in situ self-diffusion studies on amorphous Ge_*x*_Si_1–*x*_ films. In literature, no experimental data are available for the amorphous mixing system in contrast to the crystalline system.^28,29,39,40,62^

## 2 Experimental Procedure and Sample Characterization

### 2.1 Electron and Photon Based Techniques

Beneath investigations with NR for the determination of diffusivities, the samples were characterized by various methods in order to get information on the structural state. X-ray reflectometry (XRR), grazing incidence X-ray diffraction (GI-XRD), energy dispersive X-ray spectroscopy (EDX), Raman spectroscopy (RS), and Auger electron spectroscopy (AES) were performed ex situ at room temperature. XRR and GI-XRD were carried out on a Bruker D8 DISCOVER diffractometer (CuKα, 40 keV, 40 mA), with GI-XRD at an incident angle of 1°. RS was performed on a Bruker SENTERRA Raman microscope with a 532 nm laser. For further information see ref (10). EDX was performed with a high-resolution scanning electron microscope (SEM, EVO 15, Zeiss). AES spectra were measured using a NanoSAM Lab (Omicron) microscope.

### 2.2 Neutron-Based Technique

NR was performed in situ during isothermal heating of the sample in a rapid thermal annealing furnace (RTA AO600 MBE Komponenten, Germany) in argon gas. The NR study was performed on the AMOR reflectometer at SINQ, Paul Scherrer Institute, Switzerland.^52,59^ During NR, a neutron beam is directed on the sample surface at a defined angle of incidence. The neutrons are partially reflected at each interface of a layered material and all the reflected waves interfere. The interference pattern as a function of momentum transfer (the reflectivity curve) is thus a function of the layer’s indices of refraction and thicknesses.

In neutron scattering, the interaction is not electromagnetic. Neutrons interact with the atomic nucleus via the strong nuclear forces. The neutron scattering length is very short, in the low femtometer range. A discrimination of different isotopes of an element is possible. For the ^73^Ge isotope, the neutron scattering length is 5.02 fm while the neutron scattering length averaged over all Ge isotopes with natural abundance (^nat^Ge) is 8.19 fm. This gives significant contrast for the planned reflectometry experiments.

### 2.3 Sputtering Targets and Chemical Composition of Films

**Figure 1:**
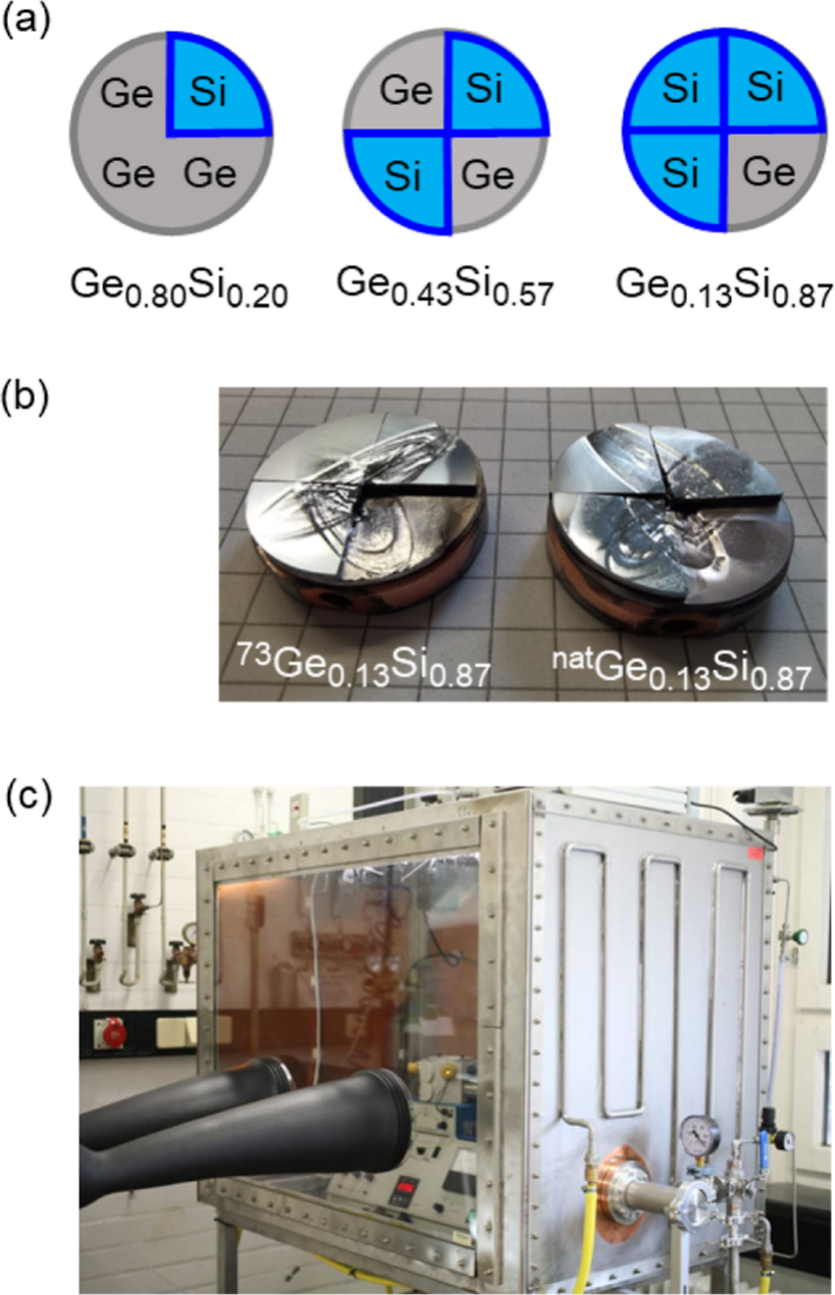
(a) Schematization of the top view of the segmented Ge–Si targets. (b) Photographs of the targets with three silicon quadrants bonded to the ^73^Ge disk (left image) and to the ^nat^Ge disk (right image). (c) IBC device under protective argon gas overpressure in a home-built glovebox with water-cooled copper walls.

The thin film samples under investigation are produced by ion-beam sputtering (see next section). The Ge–Si targets used to sputter-deposit the amorphous Ge_*x*_Si_1–*x*_ films are shown in Figure 1a. Segmented targets were used to tailor the composition. The films are deposited as isotope multilayers (MLs) {[^73^Ge_*x*_Si_1–*x*_ (≈14 nm)/^nat^Ge_*x*_Si_1–*x*_ (≈14 nm)] × 10} on polished copper foils, polished sapphire wafers (CrysTeck GmbH, Berlin, Germany), or Ge and Si wafers (MaTeck GmbH, Jülich, Germany). Photographs of characteristic targets are shown in Figure 1b. One mm thick polycrystalline ^nat^Ge or^73^Ge wafers with 20 mm diameter (MaTeck GmbH, Jülich, Germany) were bonded to a copper target holder to get films with *x* = 1 (pure Ge). The ^73^Ge target has a ^73^Ge isotope enrichment of 95%. ^73^Ge is a stable germanium isotope and ^nat^Ge refers to germanium with natural isotope abundance. To fabricate Ge_*x*_Si_1–*x*_ films with *x* < 1, quadrants of Si wafers (MaTeck GmbH, Jülich, Germany) of 1 mm thickness and 20 mm diameter were bonded to the Ge wafers to produce segmented targets as shown in Figure 1a,b. A two-component epoxy (Chemtronics, CW2400 CircuitWorks Conductive Epoxy) with excellent electrical conductivity (resistivity less than 0.001 Ω cm), rapid room temperature cure, excellent chemical and moisture resistance, and ability to bond dissimilar surfaces was used for bonding.

The chemical composition of the deposited films was measured by EDX and AES. For AES, the native oxide film at the surface of Ge_*x*_Si_1–*x*_ films was removed by sputtering. Both spectroscopy methods gave the same chemical composition within error limits. ^73^Ge and ^nat^Ge targets used to sputter the pure Ge films were modified to segmented targets in order to sputter Ge–Si films. First, only one silicon quadrant was bonded on the top of the ^73^Ge and ^nat^Ge targets (Figure 1a). Sputtering produced homogeneous films with a relative amount of (80 ± 5) at % Ge and (20 ± 5) at % Si (termed Ge_0.80_Si_0.20_). The adding of further silicon quadrants produced films with a relative amount of (43 ± 5) at % Ge and (57 ± 5) at % Si (termed Ge_0.43_Si_0.57_), and of (13 ± 5) at % Ge and (87 ± 5) at % Si termed Ge_0.13_Si_0.87_. Figure 1b shows images of the segmented ^73^Ge and ^nat^Ge targets, each with three silicon quadrants. EDX studies of the films deposited from the segmented targets give the same chemical composition for ^73^Ge_*x*_Si_1–*x*_ and ^nat^Ge_*x*_Si_1–*x*_ films within error limits. Note that the ratio of Ge to Si in the deposited films is not the same as in the sputtering target.

**Figure 2:**
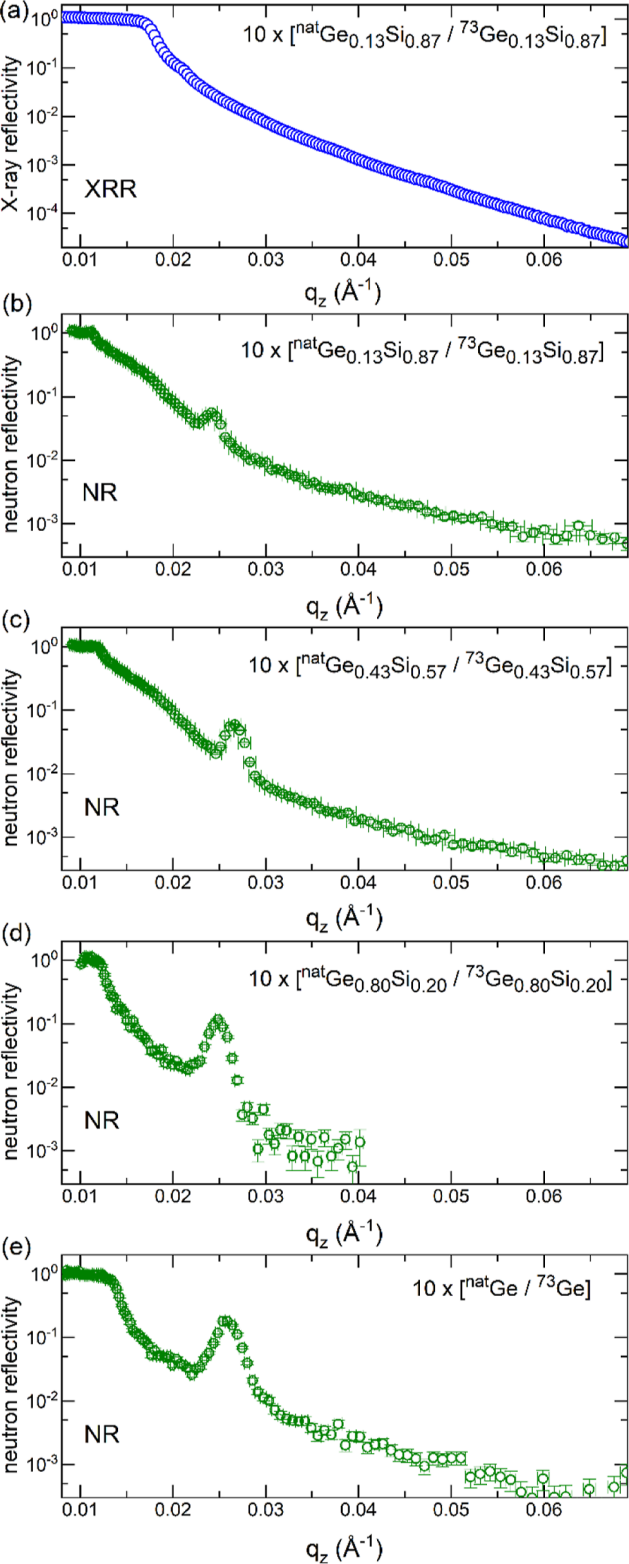
(a) XRR and NR of (b) [^73^Ge_0.13_Si_0.87_/^nat^Ge_0.13_Si_0.87_] × 10, (c) [^73^Ge_0.43_Si_0.57_/^nat^Ge_0.43_Si_0.57_] × 10, (d) [^73^Ge_0.80_Si_0.20_/^nat^Ge_0.80_Si_0.20_] × 10, and (e) [^73^Ge/^nat^Ge] × 10 ML films.

Figure 2a shows the XRR measurement of a [^73^Ge_0.13_Si_0.87_/^nat^Ge_0.13_Si_0.87_] × 10 ML film on a polished silicon wafer deposited from the targets shown in Figure 1b. The absence of Bragg peaks in the XRR pattern further proves that the layers have the same chemical composition. XRR cannot identify the Ge isotope modulation in the ML films, only chemical contrast. On the contrary, the neutron scattering length contrast between ^73^Ge (5.02 fm) and ^nat^Ge (8.19 fm) allows one to detect the Ge isotope modulation in the ML film by NR. Figure 2b shows a small Bragg peak located at a scattering vector of approximately 0.025 Å^–1^, which appears due to the Ge isotope modulation. Increasing the Ge in the films content increases the isotope contrast and the NR Bragg peak (Figure 2b–e).

### 2.4 Film Deposition

Sputter deposition of the ML was performed using a sputter coater (IBC 681, Gatan, USA) equipped with two Penning sources. Ar^+^ ion beams (5 kV, 180 μA) were used for film deposition. The targets were successively changed without breaking the vacuum with a base pressure below 5 × 10^–7^ mbar. During deposition, the sample is rotated (30 rotations per minute) and rocked (rocking angle: 30° and rocking speed: 15° per second) to ensure well dispersed Ge and Si atoms in the deposited film. (100) oriented, polished, nominally undoped silicon wafers (CrysTec GmbH, Berlin, Germany) were used as substrates for the MLs prepared for XRD, XRR, NR and AES measurements. For RS and EDX, the films were deposited on (0001) oriented polished sapphire wafers (CrysTec GmbH, Berlin, Germany) and on polished Cu foils, respectively. The substrates were cleaned with isopropanol. The deposition rate was determined by XRR measurements as described in ref (58). The as-deposited multilayers have a total thickness of around 280 nm. According to the literature, the self-heating of the sample during ion beam sputtering experiments is generally low (below 80 °C) due to the low impact energy (tens of eV) of the deposited ions.^44^ The native oxide layer of the substrates was not removed.

To minimize film contamination during sputter deposition, the IBC device was placed under argon overpressure in a custom-built glovebox with water-cooled copper walls (Figure 1c). AES measurements showed that the oxygen contamination is below the AES sensitivity limit of about 1 at % for Ge and Ge_0.80_Si_0.20_ film deposition. Oxygen contamination increases with the addition of further silicon to about 2 at % for the Ge_0.43_Si_0.57_ films and to about 5 at % for the Ge_0.13_Si_0.87_ films. The near absence of oxygen contamination in amorphous pure germanium and germanium rich layers can be explained by the higher affinity of oxygen to silicon than to germanium.

### 2.5 Amorphous State of the Films

**Figure 3:**
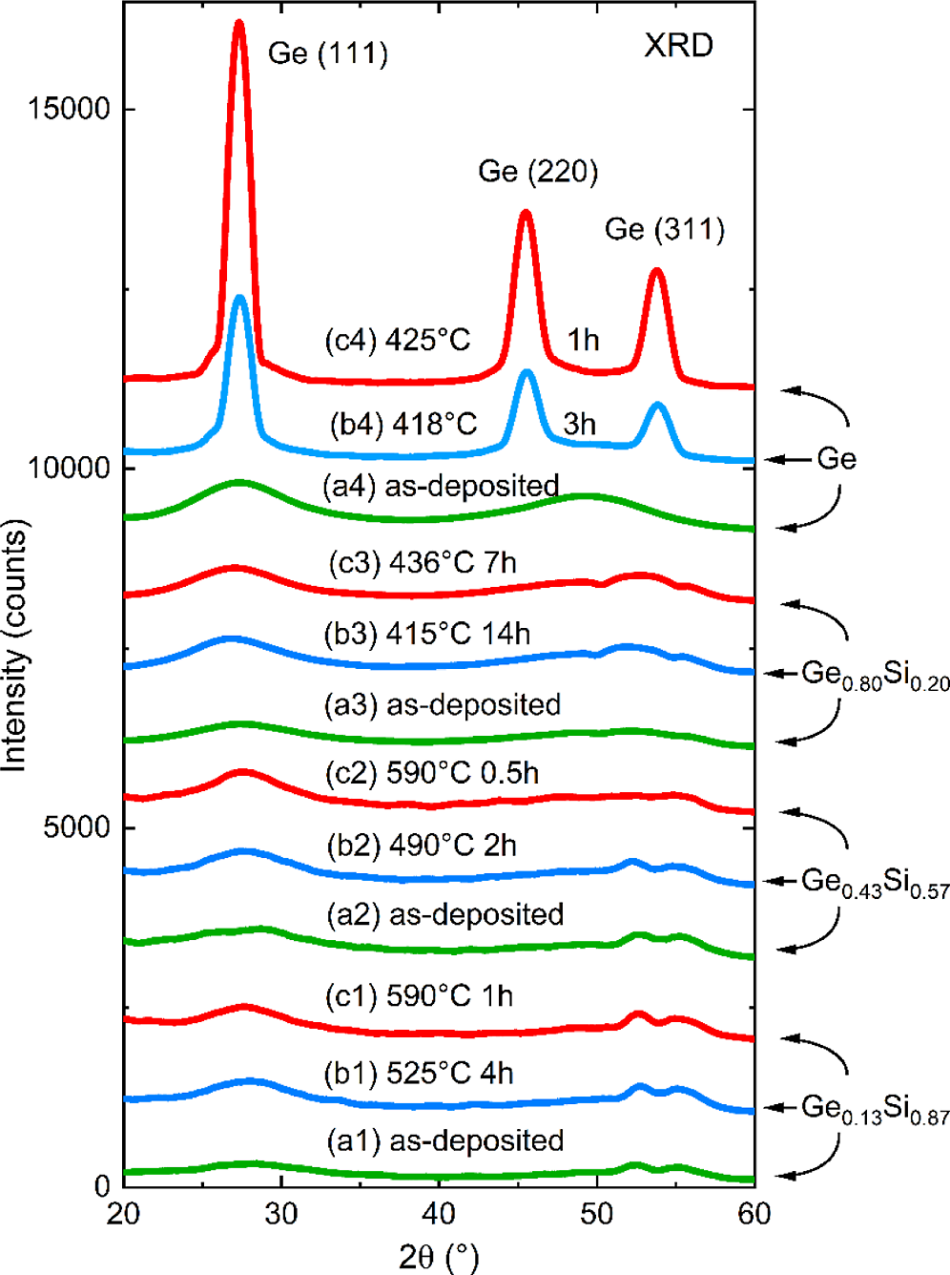
GI-XRD data of Ge_0.13_Si_0.87_ isotope ML (a1,b1,c1), Ge_0.43_Si_0.57_ isotope ML (a2,b2,c2), and Ge_0.80_Si_0.20_ isotope ML (a3,b3,c3) compared to that of pure Ge isotope ML films (a4,b4,c4). The diffraction patterns are shifted for clarity.

Figure 3 shows GI-XRD data measured before and after the thermal treatments. All as-deposited Ge_*x*_Si_1–*x*_ films are X-ray amorphous (a1–a4) and show the presence of local order only. Heat treatment of pure Ge ML at 418 °C for 3 h (b4) and at 425 °C for 1 h (c4) results in crystalline germanium. This is not the case for *x* < 1 in amorphous Ge_*x*_Si_1–*x*_ films (a2–a3, b2–b3, c2–c3), even for treatments at higher temperatures such as 590 °C. The XRD studies clearly show that the presence of silicon inhibits the crystallization of amorphous Ge_*x*_Si_1–*x*_ films compared to pure Ge films, in agreement with previous studies.^63,64^ Thus, the diffusion experiments performed in this work were carried out on X-ray amorphous Ge_*x*_Si_1–*x*_ films.

**Figure 4:**
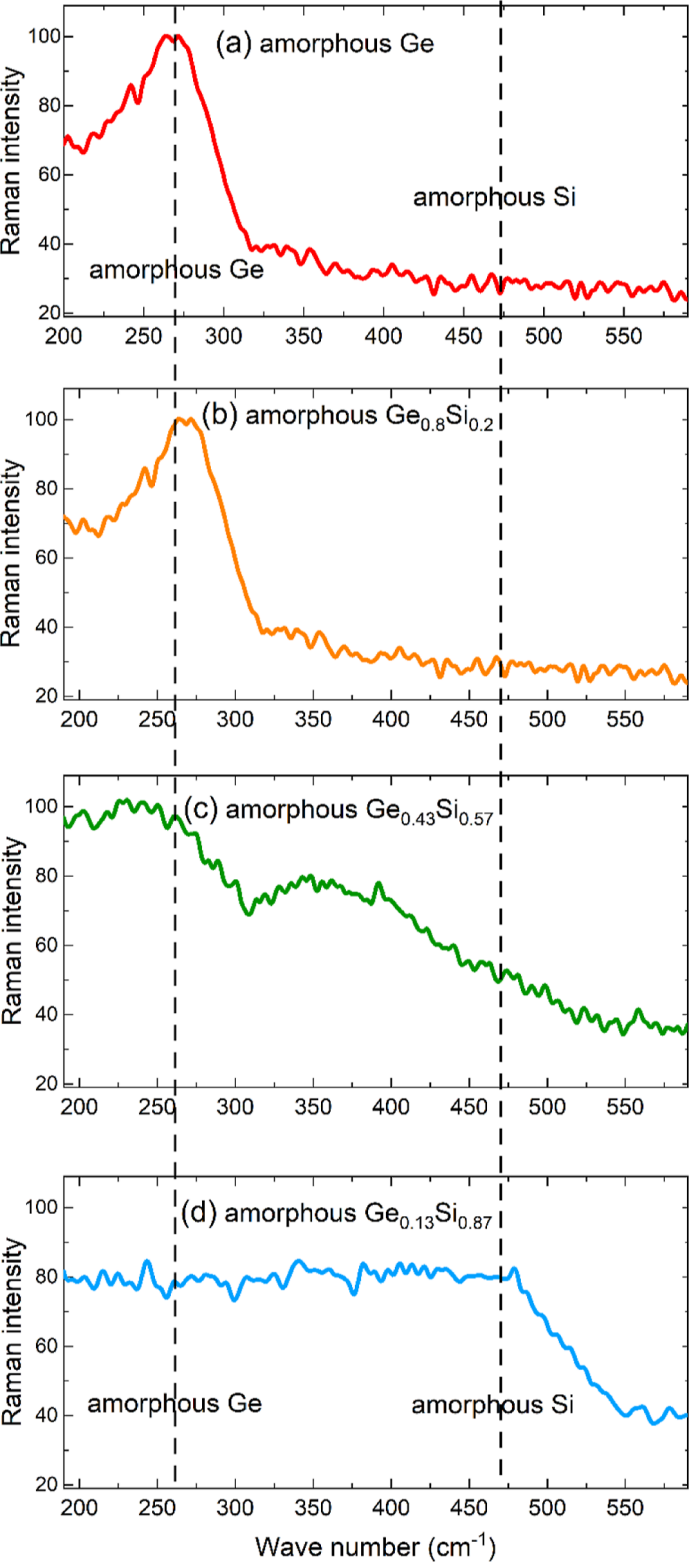
Raman spectra of amorphous (a) Ge, (b) Ge_0.80_Si_0.20_, (c) Ge_0.43_Si_0.57_ and (d) Ge_0.13_Si_0.87_ films sputter deposited on a sapphire substrate.

Figure 4 presents the RS data of the Ge_*x*_Si_1–*x*_ films. The dashed vertical lines show the wavenumber positions of the Raman bands for amorphous germanium and amorphous silicon as found in the literature.^65−67^ Ge–Si local bonding produces Raman lines between the pure materials.^65−67^ In the pure Ge film there are no Si–Si bonds (Figure 4a), as expected. This is also the case for the Ge_0.80_Si_0.20_ films (Figure 4b). The Si atoms are well dispersed in the amorphous germanium matrix. In the case of a higher silicon content of amorphous Ge_0.43_Si_0.57_ (Figure 4c) and Ge_0.13_Si_0.87_ (Figure 4d) films, there is also scattered Raman intensity at the position of pure amorphous silicon. The higher the Si content, the higher the Raman intensity at the position of Si–Si bonds. This means that there are Si atoms with Si nearest neighbor atoms due to the higher Si content. The Raman intensity is also enhanced between the peaks of the pure amorphous films without producing distinct peaks (Figure 4c,d). This means that these amorphous films have an atomic environment with Si and Ge atoms as nearest neighbors. Overall, the GI-XRD and RS measurements revealed that the Ge self-diffusion studied in this work takes place within an amorphous matrix with well dispersed Si and Ge atoms in a Ge_*x*_Si_1–*x*_ network.

## 3 In Situ NR Experiments: Results and Discussion

**Figure 5:**
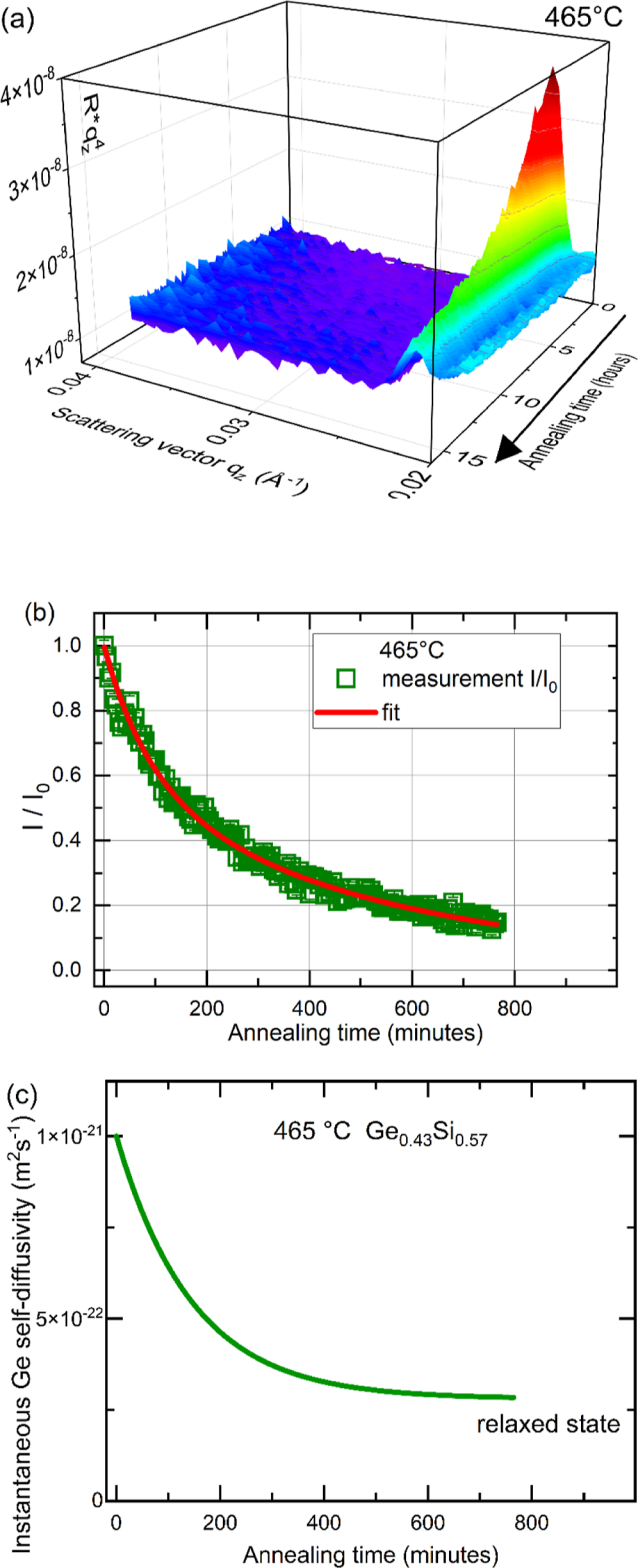
(a) In situ measured NR reflectograms during isothermal annealing at 465 °C of an amorphous Ge_0.43_Si_0.57_ isotope ML shown as a three-dimensional color map of (Rq_*z*_^4^,q_*z*_) representation. (b) Bragg peak normalized to the initial intensity value (*I*/*I*_0_) versus annealing time. The fit (red line) was performed using eq 5 with the following result: *D*_i_ = (1.0 ± 0.3) × 10^–21^ m^2^ s^–1^, *D*_R_ = (2.8 ± 1) × 10^–22^ m^2^ s^–1^, and τ = (2.4 ± 1.1) h. (c) The obtained modification of the time dependent instantaneous Ge self-diffusivity.

All types of isotope multilayers were isothermally annealed at various temperatures in range between 370 and 590 °C. The annealing leads to a decrease of the Bragg peaks with time as exemplarily illustrated in Figure 5. For certain temperatures, where the decrease of the Bragg peak is in the minute to hour range a reliable analysis was possible. Figure 5a show a typical three-dimensional color map of the in situ measured NR data in the Rq_*z*_^4^ representation during isothermal annealing. Figure 5b shows the normalized Bragg peak intensity (the integrated area of the Bragg peak) as a function of annealing time. The Bragg peak intensity decreases due to the interdiffusion of Ge isotopes.

Ge self-diffusivities can be determined from the decrease of the integrated Bragg peak intensity by the following equation
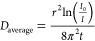
1assuming that the diffusivity, *D*_average_, is constant during the heat treatment. *I*_0_ and *I* are the Bragg peak intensities before annealing and at time *t* of the annealing process, respectively. The bilayer thickness is given by *r*. The application of this formalism give inappropriate results as also demonstrated in ref (44). Since the diffusivity changes during annealing because the annealing process changes the point defect densities, eq 1 is no longer valid and eqs 2–5 must be used,^44,46^ which give the average diffusivity over the time interval *t*. This analysis is more appropriate for amorphous films where the annealing process may change the point defect densities, e.g. frozen-in defects produced by the sputter deposition process.^44^ In this case, the average diffusivity for a given time interval *t* is
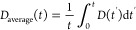
2where *D*(*t*) is the instantaneous diffusivity at time *t*, described by (first order relaxation process)
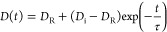
3where *D*_R_, *D*_i_ and τ are the diffusivity in the relaxed state and in the initial state (*t* = 0), and the time constant of the relaxation process, respectively. The integration of eq 3 (see refs (44 and 46)) leads to the following expression
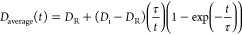
4

The expression for the intensity decrease of the Bragg peak is obtained by combining eqs 1 and 4 to get
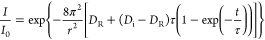
5

Equation 5 was used to fit the Bragg peak decay (see Figure 5b) with *D*_R_, *D*_i_, and τ as fit variables. The annealing time dependence of the instantaneous diffusivities, *D*(*t*), is plotted in Figure 5c. The decrease in diffusivities is within 1 order of magnitude.

**Figure 6:**
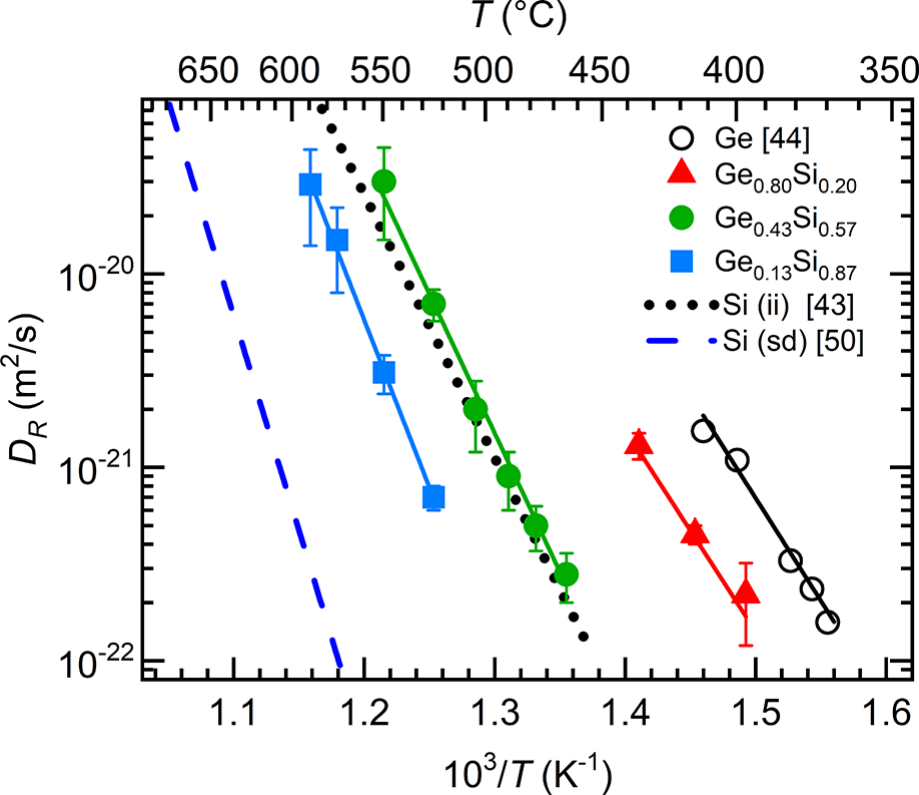
Arrhenius plot of Ge self-diffusivities (*D*_R_) in relaxed amorphous Ge_0.13_Si_0.87_ films (blue-filled squares), Ge_0.43_Si_0.57_ films (green dots), and Ge_0.8_Si_0.2_ films (red triangles) obtained during isothermal annealing and in situ NR experiments. Also shown are Si self-diffusivities in amorphous silicon produced by sputter deposition [blue dashed line (sd)]^50^ and ion-implantation [dotted black line (ii)]^43^ as well as Ge self-diffusivities in sputter-deposited amorphous relaxed germanium (black circles^44^). The straight lines correspond to fits according to eq 6.

**Figure 7:**
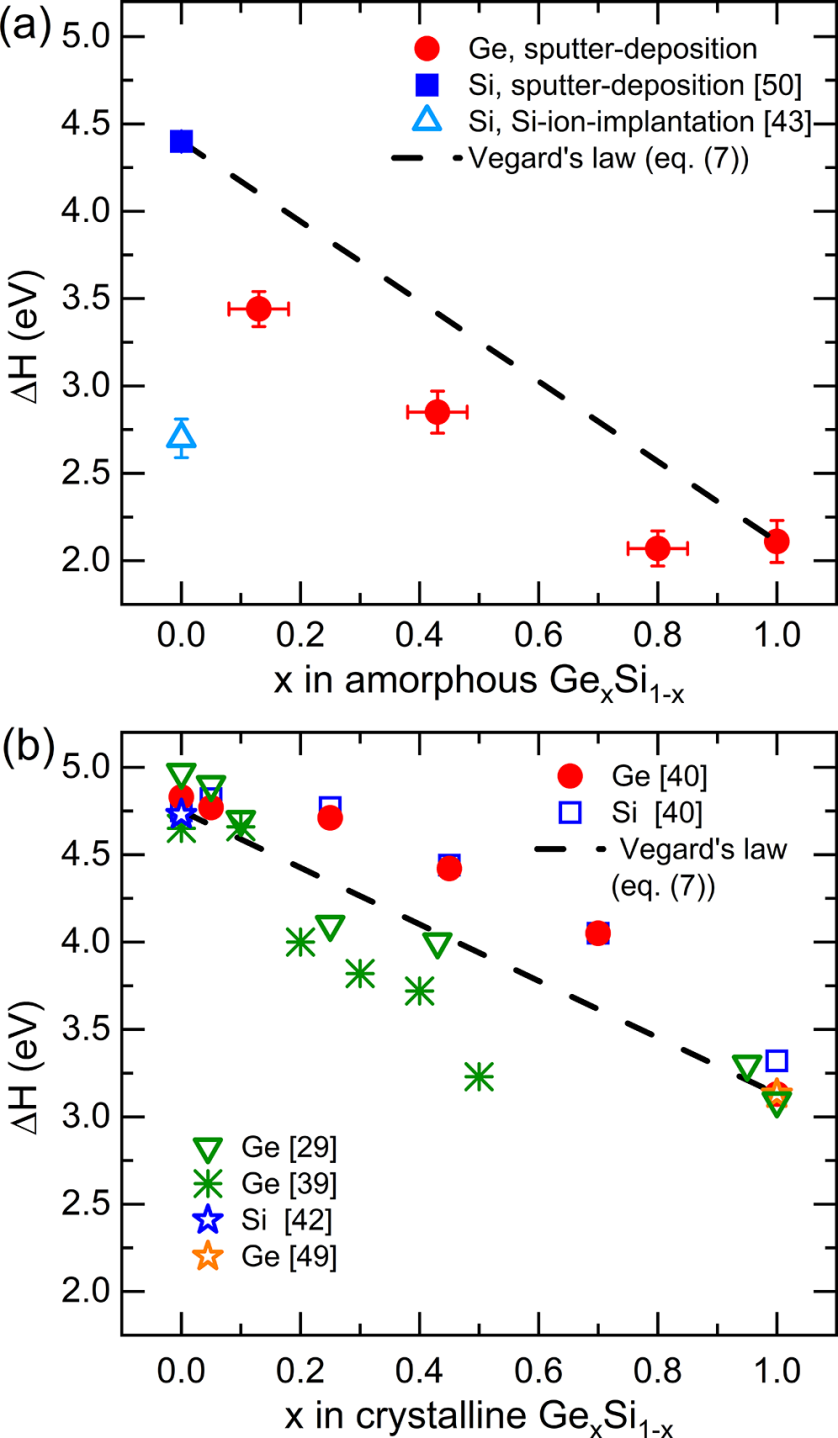
(a) Activation enthalpy (Δ*H*) of self-diffusion as a function of *x* in amorphous Ge_*x*_Si_1–*x*_. The data of Si self-diffusion in amorphous silicon produced by sputter deposition^50^ is marked with a blue filled square and that produced by Si-ion implantation^43^ with an open triangle. The activation enthalpy of Ge self-diffusion in amorphous germanium^44,51,57^ and that in amorphous Ge_0.8_Si_0.2_,^58^ Ge_0.43_Si_0.57_ and Ge_0.13_Si_0.87_ are marked with red-filled circles. (b) Activation enthalpy (Δ*H*) of self-diffusion as a function of *x* in crystalline Ge_*x*_Si_1–*x*_. The values of Si self-diffusion for pure silicon (*x* = 1)^41,42^ and of Ge self-diffusion for pure germanium (*x* = 0),^49^ are marked with a blue star and an orange star, respectively. The red dots and blue squares mark the results of Kube et al.^40^ for Ge and Si self-diffusion in epitaxial films. The green snowflakes and green triangles mark data for Ge self-diffusion in epitaxial films done by Zangenberg et al.^39^ and Strohm et al.,^29^ respectively. The expected diffusivity behavior according to eq 7 is marked with a black dashed line.

The obtained diffusivities (*D*_R_) in relaxed amorphous Ge,^44^ Ge_0.8_Si_0.2_, Ge_0.43_Si_0.57_, and Ge_0.13_Si_0.87_ are shown in Figure 6 in the Arrhenius representation. Also shown are Si self-diffusivities in pure amorphous silicon prepared by sputter deposition in our laboratory^50^ and in pure amorphous silicon prepared by Si ion-implantation.^43^ The diffusivities follow the Arrhenius law in all cases. The solid lines represent the fit of the experimentally determined diffusivities (symbols) to the Arrhenius law
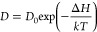
6where *D*_0_, Δ*H*, *k*, and *T* are the pre-exponential factor, the diffusion enthalpy, the Boltzmann constant, and the temperature, respectively and *D* = *D*_R_ in the present case. It is assumed that *D*_R_ represents the constant diffusivity in a well-defined relaxed metastable state. The obtained activation enthalpies and pre-exponential factors are listed in Table 1 and given in Figure 7a as a function of *x* in Ge_*x*_Si_1–*x*_. As shown in Figure 6, the Ge self-diffusivities in amorphous Ge_0.8_Si_0.2_, Ge_0.43_Si_0.57_, and Ge_0.13_Si_0.87_ are intermediate between the self-diffusivities of sputter-deposited amorphous pure Ge and Si films. The higher the Si content of a sample, the slower is the Ge self-diffusion at a given temperature.

**Table 1:** Activation Enthalpy (Δ*H*) and Pre-exponential Factor (*D*_0_) of Self-Diffusion in Amorphous Ge_*x*_Si_1–*x*_^a^

*x* in amorphous Ge_*x*_Si_1–*x*_	Δ*H*/eV	log_10_ (*D*_0_/m^2^ s^–1^)
*x* = 1	2.11 ± 0.12	–5.23 ± 1.0
*x* = 0.80	2.07 ± 0.10	–6.20 ± 1.0
*x* = 0.43	2.85 ± 0.12	–2.15 ± 0.8
*x* = 0.13	3.44 ± 0.10	0.57 ± 0.4
*x* = 0	4.40 ± 0.30	4.17 ± 1.5

a Produced by sputter-deposition.

Although the Ge self-diffusivities in Ge_*x*_Si_1–*x*_ are consistent with respect to sputter-deposited pure Si and Ge, it is not the case for self-diffusivities obtained for amorphous silicon produced by Si-ion implantation (the black dotted line in Figure 6). This indicates that amorphous structures with different diffusion properties are formed using the two preparation methods. It can be assumed, that the impurity contamination level of amorphous silicon (mainly oxygen) produced by Si-ion implantation is lower than that of samples produced by sputter deposition. Ion implantation can also produce a higher degree of disorder at the microscopic level with a higher amount of defects such as dangling bonds and probably vacancies.

Figure 7a shows the activation enthalpy (Δ*H*) of Ge self-diffusion in amorphous Ge_*x*_Si_1–*x*_ as a function of the relative Ge content *x*. All activation enthalpies are below the straight line corresponding to Vegard’s law^40^
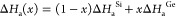
7which describes the modification of the activation enthalpy of diffusion in Ge_*x*_Si_1–*x*_ with a linear interpolation between Si and Ge. This means that for a given Ge concentration *x*, a lower activation enthalpy is measured compared to the ideal case. For the high Ge content alloy Ge_0.8_Si_0.2_, an activation enthalpy of (2.07 ± 0.10) eV was obtained from the experiments. The value is identical to that of pure amorphous Ge (2.11 ± 0.12) eV within error limits. This indicates that the diffusion mechanism of Ge in amorphous Ge_0.8_Si_0.2_ and Ge is similar, despite of the presence of dispersed 20 at % of Si. The existence of a percolation path of domains with Ge–Ge bonds is very likely. For Ge_0.13_Si_0.87_ and Ge_0.43_Si_0.57_ the activation enthalpies increase significantly with lower Ge fraction *x*, presumably due to the fact that an increasing amount of Si–Ge bonds are formed and a Ge–Ge percolation path does no longer exist. The highest activation enthalpy is found for pure amorphous silicon.

The negative deviation of the activation enthalpies from Vegard’s law may be explained with the assumption that the Ge_*x*_Si_1–*x*_ alloys consist of nanoscopic domains with different Ge concentrations. In domains with a higher Ge content the Ge diffusivity is faster than in domains with a lower Ge content and also has a lower activation enthalpy. Premise is that such Ge-rich domains form a percolation path. This means in an alloy with a formal Ge fraction of *x*, diffusion takes place along Ge richer domains with fraction *x* + δ*x*. This would place the activation enthalpy more closely to Vegard’s law.

Another plausible reason for the deviation of the activation enthalpy from Vegard’s law might be due to the presence of heterobonds (i.e., Ge–Si) that do not exist in pure germanium and pure silicon. It is very likely that with the nonlinear behavior of the bond lengths and lattice parameters in Ge_*x*_Si_1–*x*_,^68^ the formation and migration enthalpies exhibit also deviation from Vegard’s law.^40^

It should be noted that the diffusion mechanism in amorphous semiconductors and also Ge_*x*_Si_1–*x*_ is far from clear, and under discussion. If it is assumed that for the amorphous case, diffusion proceeds via a local bond break mechanism (see below), our results suggest that this mechanism may take place in an easier way with increasing Ge content. This is reflected in a decrease of the activation enthalpy and a simultaneous strong decrease of the pre-exponential factor (Table 1).

For further discussion we compare now the present results on the amorphous system to diffusivities in crystalline Ge_*x*_Si_1–*x*_ obtained from literature as shown in Figure 7b. Recent work by Kube et al.^40^ reports that the Si and Ge diffusion coefficients in crystalline pure silicon agree within experimental accuracy. However, with increasing Ge content the diffusion of Ge becomes slightly faster than that of Si.^40^ Self-diffusion in amorphous Ge and Si is faster than in their crystalline counterparts due to lower activation enthalpies. For amorphous silicon (sputter deposition), experiments found an activation enthalpy of (4.4 ± 0.12) eV,^50^ which is only slightly lower than for epitaxial (i.e., crystalline) Si films of (4.73 eV ± 0.03) eV.^41,42^ The situation is different for germanium. For amorphous germanium (sputter deposition) experiments found a lower activation enthalpy of (2.11 ± 0.12) eV^44,51,57^ instead of (3.13 ± 0.03) eV. This may suggest different diffusion mechanisms in crystalline and amorphous modifications.

Overall, the general trend of the activation enthalpies of self-diffusion in amorphous Ge_*x*_Si_1–*x*_ (Figure 7a) is similar to that in crystalline Ge_*x*_Si_1–*x*_ (Figure 7b). For both systems, there is a decrease with increasing Ge content *x*. This is the same trend as observed for the crystallization temperature of amorphous Ge_*x*_Si_1–*x*_, which decreases from 650 to 450 °C with increasing Ge content *x*.^63,64^ It is known that crystallization requires higher temperatures in Si than in Ge.^64^

According to the actual state of research, self-diffusion occurs via self-interstitials in crystalline silicon^42^ and via single vacancies in crystalline germanium.^49^ For crystalline Ge_*x*_Si_1–*x*_ alloys, the reported decrease of the activation enthalpy with increasing Ge content is explained by a reduction of the vacancy formation energy in ref (39). Above *x* = 0.1, the self-interstitial mechanism (present for pure silicon) with its high activation enthalpy is no longer favorable for diffusion, and diffusion will occur predominantly through vacancies.^39^ The data reported by Zangenberg et al.^39^ on the crystalline system show also a negative deviation from Vegard’s law as observed in the present study for the amorphous system. The activation enthalpy of self-diffusion for *x* ≥ 0.5 is equal to that of pure germanium,^39^ implying that the vacancy formation energy must be constant and equal to that of pure germanium.^39^ Consequently, the diffusion paths chosen by the Ge atoms is predominantly Ge-like already for *x* ≥ 0.5.^39^ This is similar to what is observed in the present study for *x* ≥ 0.8 for the amorphous system. In the study of Strohm et al.^29^ the Ge self-diffusion is tentatively attributed to interstitial diffusion up to *x* = 0.25 and vacancy mediated above *x* = 0.25. However, the authors of ref (40) observe a positive deviation from Vegard’s law. This indicates that an understanding of the crystalline system is far from completed and possibly depend on details of sample preparation and experimental performance.^40^

It should be noted that theoretical treatments of self-diffusion in Ge_*x*_Si_1–*x*_ is scarce, and the effect of the lack of long-range order, i.e. the amorphous atomic network, is even more. Recent computer calculations^60,61,69^ suggest that although the local order of amorphous silicon is close to that of crystalline silicon, the energetics of defect formation^62,65^ and the diffusion mechanism^69^ are very different. The formation energy of point defects such as vacancies, self-interstitials and dangling bonds is negative.^60,61^ This means that the formation of defects in the amorphous state is spontaneous and does not require thermal energy, unlike to the situation in the crystalline state. This would also explain the lower activation enthalpies in the amorphous state. The self-diffusion mechanism in amorphous silicon is also predicted by calculations to be different from that in crystalline material because well-defined elemental jump lengths do not exist in the amorphous network.^69^ Self-diffusion should proceed by atomic bond rearrangement.^69^

As a perspective, we refer to the effect of impurities on the self-diffusion mechanism in amorphous Ge_*x*_Si_1–*x*_. A future study of impurity diffusion in amorphous Ge_*x*_Si_1–*x*_ may provide a targeted clarification of the self-diffusion mechanism, as has been done for crystalline Ge_*x*_Si_1–*x*_.^39,70−73^ Sb diffusion is expected to be vacancy driven all the way from Si to Ge.^39,70−73^ In contrast, phosphorus and boron should only diffuse through interstitials.^73^ Thus, the diffusion of these species in amorphous Ge_*x*_Si_1–*x*_ may contribute to the elucidation of the self-diffusion mechanism.

## 4 Summary

The aim of the present work was to investigate the Ge self-diffusion in amorphous Ge_*x*_Si_1–*x*_ as a function of Ge content. Ge_*x*_Si_1–*x*_ films with thicknesses of about 300 nm and Ge contents of *x* = 0.13, 0.43, 0.80, and 1.0 were sputter deposited using an ion beam coater. GI-XRD and Raman studies revealed an amorphous network of well dispersed Ge atoms within the Ge_*x*_Si_1–*x*_ film. Ge self-diffusion was studied in situ by NR. For this purpose, Ge-isotope multilayers of 10 × [^nat^Ge_*x*_Si_1–*x*_(≈14 nm)/^73^Ge_*x*_Si_1–*x*_(≈14 nm)] were prepared. The layers of ^nat^Ge_*x*_Si_1–*x*_ have the natural isotopic abundance of Ge, while the layers of ^73^Ge_*x*_Si_1–*x*_ have 95% ^73^Ge isotope enrichment. The isotope contrast produces a Bragg reflex in NR patterns. Annealing at elevated temperatures promotes Ge self-interdiffusion of the isotopes, which reduces the intensity of the NR Bragg reflex. The decay rate of the NR Bragg reflex intensity is correlated to the Ge diffusion coefficient, which can be determined using appropriate models.

The Ge self-diffusivities for all investigated Ge_*x*_Si_1–*x*_ film compositions obey the Arrhenius law. With increasing Ge content in Ge_*x*_Si_1–*x*_, the activation enthalpy of diffusion and the pre-exponential factor decrease and the diffusivities increase. The activation enthalpy is below the theoretical values expected from a linear interpolation between pure germanium and pure silicon (Vegard’s law). We attribute this to the presence of mixed Si–Ge bonds and specific domain structures.
